# A new paradigm on health care accountability to improve the quality of the system: four parameters to achieve individual and collective accountability

**DOI:** 10.7189/jogh.07.010301

**Published:** 2017-06

**Authors:** Umberto Genovese, Sara Del Sordo, Gabriella Pravettoni, Igor M. Akulin, Riccardo Zoja, Michelangelo Casali

**Affiliations:** 1 Healthcare Accountability Lab, University of Milan, Milan, Italy; 2 Institute of Legal Medicine, University of Milan, Milan, Italy; 3 Department of Oncology and Hemato–Oncology (DIPO), University of Milan, Milan, Italy; 4 Department of Biomedical Sciences for Health, University of Milan, Milan, Italy; 5 European Institute of Oncology, Milan, Italy; 6 Department of Health Organization, Saint Petersburg State University, Saint Petersburg, Russia

Healthcare systems the world over are facing significant financial pressures and growing demands for services. Many nations have therefore set common goal of improving the population’s health, the quality of the outcomes, and the containment of costs [1].

A recent perspective considers health care systems as “high reliability organizations” (HROs), which are complex systems operating in a *high–stress environment* without losing sight of the objective to provide high quality results while still focusing on the assessment and management of risks [2].

So, the growing demand among patients for increasingly high quality treatments, the obligation to reduce adverse events in health care, the need for transparency in health care systems, and the current economic situation compound the difficulties in improving health care delivery. The debate on these issues now transcends national borders and single organisational, political and jurisprudential systems. Also, the problem of escalation of litigation in health care is applicable to all physicians regardless of age, geographical origin, and specialisation and it should be solved.

Therefore, these changes in the health care systems’ priorities have set the ground for an interdisciplinary approach necessary to assess the activities of health care professionals and, in general, of health care systems.

## TIME IS RIPE

Increasingly, the concept of health care professional responsibility concentrates only on medical malpractice. However, health care accountability does not depend on the hypothesis of a patient’s damage claim and should instead reflect all conditions necessary for the daily delivery of high quality health care services to the system users, which constitute rational use of the economic resources.

We believe medico–legal activity should not “merely” contribute to the evaluation of other physicians’ conduct within medical malpractice, but – as other disciplines do – it may provide further reflection to stimulate the comparison between different health care professionals and to provide a valuable support to the activities of clinical risk management.

With this background, we therefore believe that the time is ripe to offer a new technical paradigm for professional accountability, valid to lead the assessment (*ex post*) of the physicians’ conduct within the medical malpractice toward applications (*ex ante*) useful to improve the health care professionals’ approach to the system.

This paradigm is based on four distinct pillars, interlinked and interdependent.

## PROFESSIONAL COMPETENCE AND INFORMATION ACCESSIBILITY

The first parameter is *competence*, defined by Epstein and Hundert as *“*the habitual and judicious use of communication, knowledge, technical skills, clinical reasoning, emotions, values and reflection in daily practice for the benefit of the individual and community being served*”* [3]. This is necessarily a progressive acquisition involving some selective stages formed not only by theoretical knowledge, know–how and self–management skills, but also by the ability to teach and pass on. To make this concept really useful, the physician should be able to document both the competence acquired and the concise quality level of his performance, since the health care system’s users require a level of performance aimed at excellence. This, on the other hand, could exclude from the system those physicians whose performances fall below the expectations required [4]. However, there is still intense debate about how best to assess and measure the competence and performance of health care professionals. Likewise, health care facilities should also be able to document the level of their performance.

This aspect draws upon the *information accessibility* parameter because, now that the doctor–patient relationship no longer focuses on a paternalistic approach but rather on a “patient–centred” one, the patient’s information health needs must be satisfied. Information accessibility should cover the competence and the performance quality of the individual physician and the health care structure. In the former case, emphasis is placed on the curriculum, the individual and team membership performance statistics, the prevailing working activity areas (particularly in the case of super–specialised physicians), any contributions to national and international clinical and scientific progress, and multidimensional feedback data (not only technical but also relational) concerning patients already treated. Whereas information accessibility within the health care facility requires the actual availability of clinical services, the ordinary staffing and diagnostic–therapeutic equipment, the waiting times’ average values to access the services, the general and sectoral performance statistics, the overall and sectoral accident rate statistics, any scientific and academic collaborations, and multidimensional feedback data related to patients already treated and transited users. Sharing such information with service users could furthermore encourage a “healthy” competition amongst those providing the treatment (physicians or health care facilities) and this, in turn, could stimulate the continuous quality improvement of the services provided.

## AWARENESS AND GRATIFICATION

Another parameter to be taken into account is the *awareness*, intended as the consciousness every physician should have about the significance and the implications of each medical act, not only with a view to patient health protection, but also toward the health care facility where the physician operates, any insurance company, and the entire health care system. This aspect also includes the judicious use of available instrumentation and equipment so as to achieve the maximum performance not only economically, but also in terms of management and system optimization. The awareness of each medical act’s implications could also help reduce the phenomenon of defensive medicine [5].

Lastly, the final assumption of the paradigm presented here is the health care professional’s *gratification* that, though it may seem a matter of little interest, is actually a crucial requirement to maintain motivated professional behaviour. Generally until a few years ago physicians were satisfied with their career path though some dissatisfaction was derived from salary, the time spent at work, the administrative–bureaucratic aspects of daily practice, and the reduction of professional autonomy [6].

At this stage, leaving the economic aspect specifically aside, the desirable physician’s gratification should include the explicit recognition of the objective professional value of the individual doctor within the hospital or the system where he/she operates and also the guarantee of a merit–based tasks’ hierarchy (thus recalling the *competence* acquiring process): this would result in the physician’s perception of the trust patients and the entire health care system are placing in his/her work, and would lead to a higher satisfaction of the professional himself. The climate of mistrust directed towards the medical profession also implies that the physicians’ gratification should be subject to a media re–accreditation of the entire profession.

## FOUR PARAMETERS TO ACHIEVE ACCOUNTABILITY

**Figure 1:**
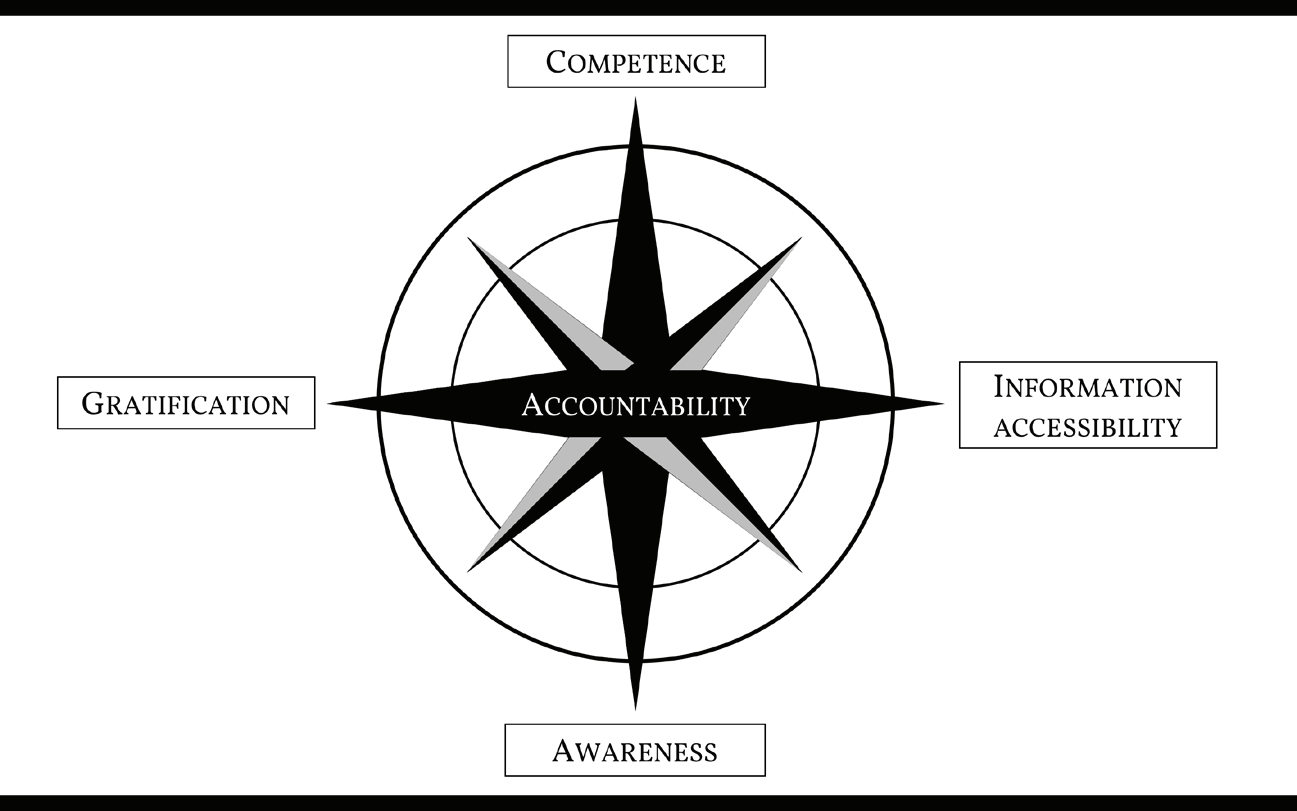
Four parameters to achieve health care accountability.

The development of each of the four proposed parameters (*competence, information accessibility, awareness and gratification*) (**Figure 1**) and their combination would allow better achievement to the coveted target of *accountability* within health care, as outlined decades ago [7,8].

Accountability was originally established as an individual professional’s domain but, alongside the evolution towards a complex health care system, it has necessarily extended to a collective and rather systematic level [9]. The knowledge that, currently, the system complexity determines the errors to a much greater extent than the individual professional’s behaviour, has led to a progressive re–evaluation of the concepts of responsibility and accountability. This, however, still requires a cultural change so as to ensure that the whole society comprehends that health care safety depends not only on the individual physician but also on the system and that, in case of a system failure, the individual professional is not solely responsible.

Accountability has evolved from an individual to a collective dimension, namely a concept in which all providers, in concert with health care institutions, work collaboratively to share responsibility for transparency, error prevention and ‘making the patient whole’, as defined by Bell et al. [10]. In other words, accountability is the synthesis between credibility and reliability, both of the individual professional, and the institutions and the health care system. On the one hand the individual physician acquires the tools to adequately respond to the patient’s expectations (horizontal accountability) and to the obligations to society, which inevitably result from the position of a health care system within the society (thus distinguishing a vertical accountability). On the other hand, the health care system acquires the tools for proper management control of its professionals, with the possibility to monitor physicians’ placement in certain positions and for the appropriate time, according to their competence and level of performance.

In conclusion, we believe that the physician’s “patient–centred” orientation also involves the paradigm of the health care accountability approach previously outlined, which is consistent with the current complexity of health care systems and society’s demands.
